# *LRPPRC* mutations cause early-onset multisystem mitochondrial disease outside of the French-Canadian population

**DOI:** 10.1093/brain/awv291

**Published:** 2015-10-28

**Authors:** Monika Oláhová, Steven A. Hardy, Julie Hall, John W. Yarham, Tobias B. Haack, William C. Wilson, Charlotte L. Alston, Langping He, Erik Aznauryan, Ruth M. Brown, Garry K. Brown, Andrew A. M. Morris, Helen Mundy, Alex Broomfield, Ines A. Barbosa, Michael A. Simpson, Charu Deshpande, Dorothea Moeslinger, Johannes Koch, Georg M. Stettner, Penelope E. Bonnen, Holger Prokisch, Robert N. Lightowlers, Robert McFarland, Zofia M. A. Chrzanowska-Lightowlers, Robert W. Taylor

**Affiliations:** 1 Wellcome Trust Centre for Mitochondrial Research, Newcastle University, Newcastle upon Tyne, NE2 4HH, UK; 2 Department of Neuroradiology, Royal Victoria Infirmary, Newcastle upon Tyne, NE1 3BZ, UK; 3 Institute of Human Genetics, Helmholtz Zentrum München, Ingolstädter Landstrasse 1, D-85764 Neuherberg, Germany; 4 Institut für Humangenetik, Technische Universität München, Arcisstrasse 21, 80333 Munich, Germany; 5 Department of Biochemistry, University of Oxford, South Parks Road, Oxford, OX1 3QU, UK; 6 Willink Biochemical Genetics Unit, Manchester Centre for Genomic Medicine, Central Manchester University Hospitals NHS Foundation Trust, Manchester, M13 9WL, UK; 7 Centre for Inherited Metabolic Disease, Evelina Children’s Hospital, Guy’s and St. Thomas’ NHS Foundation Trust, London, SE1 7EH, UK; 8 Division of Genetics and Molecular Medicine, King’s College London School of Medicine, London, SE1 9RY, UK; 9 Department of Genetics, Guy’s and St. Thomas’ NHS Foundation Trust, London, SE1 9RT, UK; 10 Department of Paediatrics, University Children’s Hospital, A-1090 Vienna, Austria; 11 Department of Paediatrics, Paracelsus Medical University Salzburg, 5020 Salzburg, Austria; 12 Department of Paediatric Neurology, Georg August University, 37075 Göttingen, Germany; 13 Department of Molecular and Human Genetics, Baylor College of Medicine, Houston, TX 77030, USA

**Keywords:** *LRPPRC*, COX deficiency, mitochondrial disease, Leigh syndrome, malformations

## Abstract

Mitochondrial Complex IV [cytochrome *c* oxidase (COX)] deficiency is one of the most common respiratory chain defects in humans. The clinical phenotypes associated with COX deficiency include liver disease, cardiomyopathy and Leigh syndrome, a neurodegenerative disorder characterized by bilateral high signal lesions in the brainstem and basal ganglia. COX deficiency can result from mutations affecting many different mitochondrial proteins. The French-Canadian variant of COX-deficient Leigh syndrome is unique to the Saguenay-Lac-Saint-Jean region of Québec and is caused by a founder mutation in the *LRPPRC* gene. This encodes the leucine-rich pentatricopeptide repeat domain protein (LRPPRC), which is involved in post-transcriptional regulation of mitochondrial gene expression. Here, we present the clinical and molecular characterization of novel, recessive *LRPPRC* gene mutations, identified using whole exome and candidate gene sequencing. The 10 patients come from seven unrelated families of UK-Caucasian, UK-Pakistani, UK-Indian, Turkish and Iraqi origin. They resemble the French-Canadian Leigh syndrome patients in having intermittent severe lactic acidosis and early-onset neurodevelopmental problems with episodes of deterioration. In addition, many of our patients have had neonatal cardiomyopathy or congenital malformations, most commonly affecting the heart and the brain. All patients who were tested had isolated COX deficiency in skeletal muscle. Functional characterization of patients’ fibroblasts and skeletal muscle homogenates showed decreased levels of mutant LRPPRC protein and impaired Complex IV enzyme activity, associated with abnormal COX assembly and reduced steady-state levels of numerous oxidative phosphorylation subunits. We also identified a Complex I assembly defect in skeletal muscle, indicating different roles for LRPPRC in post-transcriptional regulation of mitochondrial mRNAs between tissues. Patient fibroblasts showed decreased steady-state levels of mitochondrial mRNAs, although the length of poly(A) tails of mitochondrial transcripts were unaffected. Our study identifies *LRPPRC* as an important disease-causing gene in an early-onset, multisystem and neurological mitochondrial disease, which should be considered as a cause of COX deficiency even in patients originating outside of the French-Canadian population.

## Abstract (precis)

The French-Canadian variant of COX-deficient Leigh syndrome (LSFC) is unique to Québec and caused by a founder mutation in the *LRPPRC* gene. Using whole exome sequencing, Oláhová *et al.* identify mutations in this gene associated with multisystem mitochondrial disease and early-onset neurodevelopmental problems in ten patients from different ethnic backgrounds.

## Graphical abstract

**Figure:**
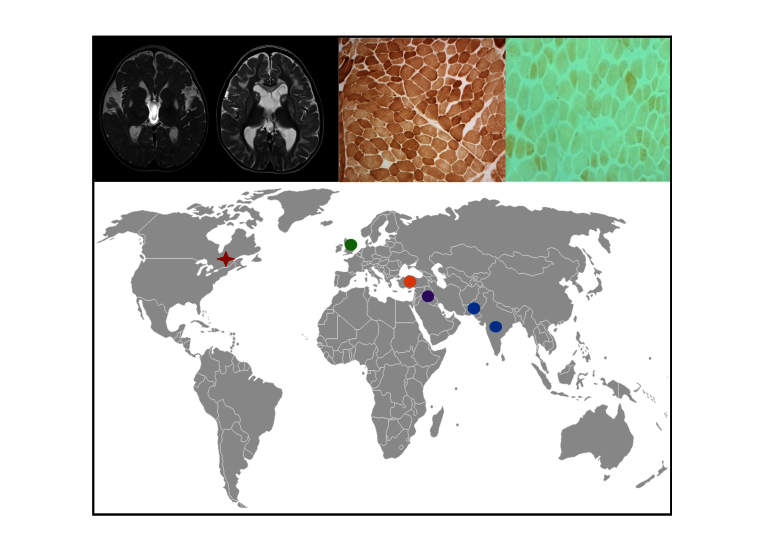
The French-Canadian variant of COX-deficient Leigh syndrome (LSFC) is unique to Québec and caused by a founder mutation in the *LRPPRC* gene. Using whole exome sequencing, Oláhová *et al.* identify mutations in this gene associated with multisystem mitochondrial disease and early-onset neurodevelopmental problems in ten patients from different ethnic backgrounds.

## Introduction

The mitochondrial oxidative phosphorylation (OXPHOS) system is the cell’s primary source of energy produced in the form of adenosine triphosphate (ATP). This metabolic pathway comprises the four transmembrane enzyme complexes (CI–IV) of the mitochondrial respiratory chain and Complex V, the F_o_F_1_-ATP synthase. A wide range of paediatric and adult-onset multisystem diseases are associated with deficiencies in the OXPHOS system affecting at least 1 in 4300 individuals (Skladal *et al.*, 2003; Gorman *et al.*, 2015). Due to the dual genetic (nuclear and mitochondrial DNA) origin of genes encoding the structural components of the mitochondrial OXPHOS system, mitochondrial respiratory chain disorders can be inherited as Mendelian traits, inherited maternally or may occur sporadically. The clinical presentation of mitochondrial disorders is associated with a wide spectrum of organ and tissue involvement and the age of onset can vary. Although the correlation between the clinical and genetic diversity of respiratory chain disorders is poor and apparent phenotype-genotype association is often not present, the biochemical assessment and characterization of the underlying OXPHOS defect underpins the molecular diagnosis of these conditions (Spiegel *et al.*, 2014).

Leigh syndrome is an early-onset subacute necrotizing encephalopathy with typical symmetrical basal ganglia and/or brainstem involvement. Developmental regression is a characteristic, but not universal feature. Leigh syndrome is commonly associated with isolated cytochrome *c* oxidase (COX) deficiency due to mutations in several COX assembly factors, including *SURF1*, which encodes a biogenesis factor participating in the early steps of Complex IV maturation, the most common cause of COX-deficient Leigh syndrome (Smith *et al.*, 2005; Bundschuh *et al.*, 2009; Wedatilake *et al.*, 2013). Mutations in other nuclear-encoded genes responsible for a generalized decrease in COX activity have been identified; these include the apoptosis regulator *APOPT1* gene (Melchionda *et al.*, 2014), the Complex IV biogenesis factor *PET100* (Lim *et al.*, 2014; Olahova *et al.*, 2015), *SCO1* and *SCO2* that are involved in copper delivery during Complex IV maturation (Leary *et al.*, 2009) and *COX10* and *COX15*, both of which have a role in the biosynthesis of mitochondrial heme *a* (Antonicka *et al.*, 2003; Oquendo *et al.*, 2004; Bugiani *et al.*, 2005). Other mutations also associated with COX deficiency in humans include *TACO1*, *COA3*, *COX14* and *LRPPRC*, encoding regulators of mitochondrial transcription and translation of transcripts encoding Complex IV subunits (Weraarpachai *et al.*, 2009; Sasarman *et al.*, 2010, 2015; Szklarczyk *et al.*, 2012; Ostergaard *et al.*, 2015).

A homozygous c.1061C>T, p.(Ala354Val) founder mutation in the *LRPPRC* gene underpins the founder French-Canadian variant of Leigh Syndrome (LSFC) identified in the Saguenay-Lac-Saint-Jean region of Québec (Mootha *et al.*, 2003). LRPPRC belongs to a family of pentatricopeptide repeat (PPR) proteins containing 35 amino acid repeat motifs that confer an ability to recognize RNA substrates and thus regulate a number of post-transcriptional processes such as RNA editing, RNA stability or RNA degradation (Lightowlers and Chrzanowska-Lightowlers, 2008, 2013; Rackham and Filipovska, 2012; Barkan and Small, 2014). While the precise molecular mechanism of the seven PPR proteins found in humans remains mostly unclear, a number of studies have identified important roles for PPR proteins in the regulation of RNA metabolism. Large mitochondrial polycistronic precursor RNAs are processed to release the 11 OXPHOS transcripts that are subsequently polyadenylated by the mitochondrial poly(A) polymerase MTPAP (Tomecki *et al.*, 2004). This process involves the addition of poly(A) tails (∼45 nucleotides) that is necessary to generate the termination codon at the 3’ end of seven of the mitochondrial transcripts (Ojala *et al.*, 1981). Recent studies in mice and humans demonstrated that LRPPRC interacts with the stem-loop RNA-binding protein SLIRP, and this complex appears to be essential for the maintenance of polyadenylated mitochondrial transcripts (Bratic *et al.*, 2011; Chujo *et al.*, 2012; Ruzzenente *et al.*, 2012; Wilson *et al.*, 2014). In addition to its role in post-transcriptional handling of mitochondrial mRNAs, LRPPRC has distinct functions in diverse cellular process; increased expression of LRPPRC has been documented in various tumours, contributing to the apoptosis resistance of human cancer cells (Tian *et al.*, 2012) and it has been identified as an inhibitor of autophagy and mitophagy via interaction with the mitophagy initiator Parkin (Zou *et al.*, 2014).

We performed whole exome and candidate gene sequencing in 10 patients from seven families with mitochondrial disease presentations characterized by infantile lactic acidosis, severely impaired psychomotor development and isolated COX deficiency. This approach identified novel homozygous and compound heterozygous mutations in the *LRPPRC* gene—the first cases to be identified outside of the Saguenay-Lac-Saint-Jean region of Québec. The levels of LRPPRC protein were decreased in patient samples and consistent with previous studies, skeletal muscle and skin fibroblast cell lines of these individuals showed a marked decrease in the steady-state levels of a number of mitochondrial mRNAs. In addition, a decrease in steady-state protein levels of Complex I and Complex IV, accompanied by abnormal Complex IV assembly, was detected in skin fibroblasts, further delineating the role of LRPPRC in the regulation of mitochondrial post-transcriptional gene expression.

## Materials and methods

### Ethics statement

This study was approved and performed under the ethical guidelines issued by each of our institutions and complied with the Declaration of Helsinki.

### Histochemical and biochemical analyses

Standard histological and histochemical analyses of diagnostic skeletal muscle biopsies, including the assay of cytochrome *c* oxidase, were performed according to established protocols on fresh-frozen sections (10 µm) (Old and Johnson, 1989). Mitochondrial respiratory chain complex activities were determined in skeletal muscle homogenates and enriched cultured skin fibroblast mitochondrial fractions as previously described, and expressed relative to the activity of the matrix marker enzyme, citrate synthase (Kirby *et al.*, 2007).

### Molecular genetic studies

Total DNA was extracted by standard procedures from available tissues obtained with consent. Copy number abnormalities and rearrangements of mtDNA were excluded by quantitative PCR and long-range PCR, respectively, whereas direct sequencing of the entire mitochondrial genome was performed on homogenate skeletal muscle DNA to exclude pathogenic mtDNA mutations. Direct sequencing of a number of candidate nuclear genes associated with isolated COX deficiency including *SURF1*, *COX10*, *COX15*, *SCO1* and *SCO2* were undertaken using standard protocols.

Whole exome sequencing was undertaken to elucidate the molecular basis of the mitochondrial disease in the probands from six families (Patients 1, 4, 5, 8, 9 and 10) using previously described methodologies and bioinformatics filtering pipelines (Haack *et al.*, 2012; Jones *et al.*, 2012; Bonnen *et al.*, 2013; Besse *et al.*, 2015). The *LRPPRC* mutation in Patient 2 was identified by candidate gene sequencing of PCR products amplified from fibroblast cDNA and confirmed by genomic DNA sequencing. All mutations were confirmed by Sanger sequencing of PCR-amplified products using BigDye® Terminator cycle sequencing chemistry (Applied Biosystems, ABI) on an ABI3130xl Genetic Analyser. Sequence data was analysed using Mutation Surveyor software v4.0.9 (SoftGenetics) and compared to the GenBank reference sequence, NM_133259.3. Mutation nomenclature is according to Human Genome Variation Society (HGVS) guidelines.

### Cell culture

Primary patient fibroblasts (Patients 1, 2 and 4) and age-matched control cell lines (Controls 1 and 2) were grown in Eagle’s Minimal Essential Medium (Sigma) supplemented with 10% (v/v) foetal bovine serum, non-essential amino acids, 1 mM sodium pyruvate and 50 μg/ml uridine at 37°C in a humidified atmosphere of 5% CO_2_.

### Micro-scale oxygraphy analysis

Live cell respiration studies were performed by micro-scale oxygraphy using the Seahorse XF^e^ Extracellular Flux Analyzer 24 (Seahorse Bioscience) according to manufacturer’s instructions and as previously described (Bonnen *et al.*, 2013; Yarham *et al.*, 2014). Briefly, patient fibroblasts were seeded at a density of 30 000 cells/well and mitochondrial function was assayed through the sequential addition of oligomycin (to 1.3 µM) to block the ATP synthase, two additions of carbonyl cyanide 4-(trifluoromethoxy)-phenylhydrazone (FCCP), a respiratory uncoupler that drives maximal respiration (to 2 µM and then to 3 µM), and antimycin A (to 2.5 µM) to inhibit Complex III.

Oxygen consumption rate (OCR) and proton production rate measurements for each well were normalized by cell number. Non-mitochondrial respiration was subtracted from all OCR values prior to analysis: spare respiratory capacity = maximal OCR − basal OCR; ATP coupling efficiency = (basal OCR − oligomycin-inhibited OCR)/(basal OCR × 100). Seven separate control cell lines underwent multiple testing to calculate control data (mean ± SD; *n* = 7), patient fibroblasts were tested multiple times as indicated in Fig. 4. An unpaired, two-tailed Student’s *t*-test was performed to determine the significance of differences between the data sets and *P*-values were considered significant at the 95% confidence interval (CI).

### Cell lysis and western blot analysis

Control and patient fibroblasts were pelleted and resuspended in lysis buffer containing 50 mM Tris-HCl pH 7.5, 130 mM NaCl, 2 mM MgCl_2,_ 1 mM phenylmethanesulphonyl fluoride (PMSF), 1% Nonidet™ P-40 (v/v) and EDTA free protease inhibitor cocktail (Pierce). Cells were lysed on ice for 30 min and lysates were clarified by centrifugation at 500 *g* for 5 min. Protein concentration was determined by Bradford method (Bio-Rad). Laemmli sample buffer containing 1% SDS, 10% glycerol, 10 mM Tris-HCl, pH 6.8, 1 mM EDTA and 50 mM dithiothreitol was added to the muscle homogenates obtained as described below. Equal amounts of each protein were loaded on 12% gels and resolved by sodium dodecyl sulphate–polyacrylamide gel electrophoresis (SDS-PAGE), followed by wet transfer to polyvinyl difluoride (PVDF) membrane (Immobilon™-P, Millipore Corporation). Immunodetection was performed using primary and horseradish peroxidise-conjugated secondary antibodies as indicated (Supplementary material).

### Mitochondrial preparation and blue native electrophoresis

Skin fibroblasts were resuspended in homogenization buffer (HB) [0.6 M mannitol, 1 mM EGTA, 10 mM Tris-HCl pH 7.4, 1 mM PMSF and 0.1 % (v/v) bovine serum albumin (BSA)] and subjected to 3 × 15 passes of homogenization using a Teflon glass Dounce homogenizer at 4°C. Mitochondria were separated from nuclei and cell debris by standard differential centrifugation (400 *g* for 10 min). Mitochondria were pelleted by centrifugation at 11 000*g* for 10 min at 4°C and washed in homogenization buffer without BSA. Similarly, muscle tissues from Patients 2 and 4 and two control subjects (∼30 mg) were homogenized using a Teflon glass Dounce homogenizer at 4°C (20 strokes) in homogenization buffer containing 250 mM sucrose, 20 mM Imidazole-HCl pH 7.4 and 1 mM PMSF. The muscle homogenates were centrifuged at 20 000*g* for 10 min at 4°C and the pellet was washed twice with 1 ml of homogenization buffer at 20 000*g* for 5 min at 4°C. The final pellets were solubilized with n-dodecyl β-D-maltoside (DDM) (Sigma) at 2 mg/mg protein on ice for 20 min. Following centrifugation at 100 000*g* for 15 min at 4°C the supernatants were retained for blue native polyacrylamide gel electrophoresis (BN-PAGE). Protein concentration was determined with the Pierce BCA Protein Assay Kit. A minimum of 30 μg of muscle and 100 µg of fibroblast protein extracts were loaded on a native PAGE 4–16 % BisTris gel (Life Technologies) and electrophoretically separated in first dimension according to the Novex® NativePAGE™ Bis-Tris Gel System instructions. Subsequently, the proteins were immobilized on PVDF membrane and subjected to standard immunoblotting analysis of OXPHOS complexes (Supplementary material).

### RNA extraction and northern blot analysis

Total RNA was extracted from fibroblasts and muscle tissues using TRIzol® reagent (Invitrogen) according to manufacturer’s instructions. RNA samples (3 µg) were loaded on 1.2% agarose gels, electrophoretically separated under denaturing conditions and subsequently transferred to a Genescreen Plus membrane (Life Science Products, Inc.). ^32^P-dCTP-labelled probes were generated from PCR products using random hexamer labelled DNA fragments. The radiochemical signal was visualized by the Typhoon FLA 9500 instrument.

### ^35^S metabolic labelling

Mitochondrial translation products in control and patients fibroblasts were pulse-labelled for 1 h with 200 µCi/ml ^35^S methionine/cysteine mix (Perkin Elmer) in Dulbecco’s Modified Eagle’s medium (Sigma) lacking methionine and cysteine. The media was supplemented with 100 µg/ml of emetine to inhibit cytosolic translation [essentially as described in Sasarman and Shoubridge (2012)]. Protein extracts (15 µg) were electrophoretically separated by 15–20% gradient SDS-PAGE and visualized using a Typhoon FLA 9500 instrument.

## Results

### Case reports

#### Patient 1 (Family 1)

This girl, the second child of second cousin parents with two healthy daughters, was born at term by normal delivery, with a low birth weight of 2320 g. She presented shortly thereafter with severe lactic acidaemia (20 mmol/l) and coagulopathy. The lactic acidaemia and coagulopathy resolved with conservative management, but she fed poorly and by 4 months her weight had fallen below the 0.4th centile. She was microcephalic and mildly dysmorphic with a prominent forehead. Psychomotor development was severely impaired: she was hypotonic and never sat independently, though at 12 months she could roll from front to back, transfer an object from hand to hand and demonstrated polysyllabic babble. Echocardiography was normal apart from mild mitral regurgitation. From 6 months of age, a chronic hyperlactataemia (4 mmol/l) was punctuated by recurrent episodes of severe lactic acidosis with ketosis, associated with vomiting and intercurrent illnesses. At 15 months, during one such episode, she developed pulmonary oedema and was ventilated, but suffered an asystolic cardiac arrest and died.

#### Patient 2 (Family 2)

This boy is the third child of first cousin parents who have two healthy daughters. He was born at term by an emergency Caesarean section for foetal distress. Antenatal ultrasound had revealed coarctation of the aorta, although this was not haemodynamically significant when assessed postnatally. By 8 months he was noted to have generalized hypotonia and was failing to thrive. Beginning at 11 months, he has had repeated hospital admissions with pneumonia, lactic acidosis (up to 18 mmol/l) and ketonuria. Between episodes, plasma lactate concentrations remained elevated (2.5–6 mmol/l) and the acidosis was managed with sodium bicarbonate. With the exception of these acute exacerbations his clinical course has been stable. He fed slowly and, from 5 years, oral feeding has been supplemented via a gastrostomy. He has shown very slow developmental progress and is still unable to sit without support at 8 years. He can manipulate and transfer objects using a palmar grasp, reaching non-preferentially with either hand. Vision and hearing appear normal, but communication is limited to crying. He is mildly dysmorphic, with a broad nasal bridge, mild hirsutism and microcephaly (<0.4%). Repeat echocardiography showed bilateral superior vena cava but no other abnormalities. Plasma amino acid analysis showed raised alanine concentrations, while urine organic acids showed increased tricarboxylic acid cycle intermediates.

#### Patient 3 (Family 2)

The younger sister of Patient 2, this girl had a birth weight of 3060 g. She presented at 20 h of age with lactic acidaemia (18 mmol/l) and hypoglycaemia (1.0 mmol/l). There have been further episodes of acidosis during illnesses, with a mild chronic lactic acidaemia, similar to her brother. She has also required treatment with regular sodium bicarbonate and tube feeding through a gastrostomy. Psychomotor development is better than her brother’s. At 2 years, she sat without support and transferred objects from hand to hand; at 3 years, she crawls and pulls to stand but she still has no words, communicating by pointing and other non-verbal means. As with her brother there has been no regression. She has a convergent squint, but vision and hearing are normal. Although not microcephalic (head circumference on 2nd centile), she has similar features to her brother with mild hirsutism and a broad nasal bridge. Cardiovascular examination and echocardiography are normal.

#### Patient 4 (Family 3)

This boy was born to healthy, non-consanguineous Caucasian parents following a normal pregnancy; his mother had a healthy boy by a different partner 11 years previously. He was born in good condition, but over the next 2 days became increasingly floppy and uninterested in feeds. He suffered several brief generalized seizures and developed a severe encephalopathy accompanied by central hypopnoea that required positive airway pressure support and supplemental oxygen. On examination he demonstrated profound hypotonia with generalized weakness and paucity of movement, but intact reflexes. There was no evidence of cardiac, renal or hepatic disease. Capillary blood gases showed a metabolic acidosis with a consistently elevated blood lactate. Lumbar puncture revealed an elevated CSF lactate (5.1 mmol/l) and low CSF glucose (1.3 mmol/l) with a decreased CSF:blood glucose ratio (0.29). The patient continued to deteriorate, developing a profound persistent metabolic acidosis and severe encephalopathy. He died following withdrawal of care at 6 weeks of age.

#### Patient 5 (Family 4)

This girl was the second child to first cousin Indian parents who have had two other affected children (Patients 6 and 7), three miscarriages and a maternal family history of Zellweger’s syndrome. She was born at term weighing 3250 g after a pregnancy that was complicated by first trimester maternal bleeding. Although initially in good condition, poor oral intake during the first 24 h was followed by hypoglycaemia and she was admitted to the special care baby unit where elevated blood lactate (16.8 mmol/l) and ammonia (118 mmol/l) were noted. Progress with feeding remained slow and she was discharged at 4 weeks, fed via nasogastric tube, although by 4 months she had been weaned onto solid food. Neurodevelopmentally she showed good progress initially, being able to sit unsupported by 7 months and to stand by 14 months. At 18 months, she developed a protracted gastroenteritic illness, following which she became increasingly floppy and lost some of her previously acquired developmental skills. Assessment at 2 years 3 months revealed that she was able to stand with support, walk holding hands, had an immature pincer grip and was able to transfer objects from hand to hand. She had no clear words but did babble and, on testing, had normal hearing. Examination revealed mild hypotonia with normal reflexes and no obvious facial asymmetry or overt dysmorphism.

#### Patient 6 (Family 4)

The older sister of Patient 5, this girl was born after induction of labour at 39 weeks weighing 2660 g. First trimester ultrasound scanning had shown increased nuchal thickness and although subsequent chorionic villus sampling confirmed a normal female karyotype, a detailed antenatal cardiac scan revealed a complex cardiac malformation with hypoplastic left heart, atrio-ventricular septal defect and aortic and mitral valve atresiae. At birth she was in good condition, but was noted to have increased nuchal folds, micrognathia, low set ears and an anteriorly placed anus. Blood lactate was elevated and skin and skeletal muscle biopsies were performed opportunistically during the first stage of cardiac surgery to correct the cardiac malformations. Although initial recovery from the procedure was uneventful, on postoperative Day 3 (Day 8 of life), blood lactate was markedly elevated (15 mmol/l), discordant with haemodynamic status. The following day the baby died suddenly after an unexplained cardiopulmonary collapse.

#### Patient 7 (Family 4)

This is the younger sister of Patient 5 who was born by spontaneous vaginal delivery at term, following an uneventful pregnancy, weighing 3600 g. She was in good condition at birth and did not require resuscitation. However, she subsequently developed intermittent tachypnoea and was transferred to the special care baby unit where she was noted to have low blood glucose and elevated blood lactate. She had a poorly co-ordinated swallow and has been fed via nasogastric tube since this time. Germinal cysts were noted on cranial ultrasound, which was otherwise normal. Blood lactate subsequently stabilized but remained mildly raised (2–3 mmol/l) at the time of her discharge home. At 9 weeks she was able to smile and she could grasp objects in her hand. Head control was considered age appropriate. She has gastro-oesophageal reflux in addition to her dysphagia but clinical examination was normal with a head circumference of 38.2 cm (25th centile).

#### Patient 8 (Family 5)

This boy was the first child of first cousin Turkish parents born at term by vaginal delivery following a normal pregnancy and weighing 2080 g with a head circumference of 30 cm. He did not require immediate resuscitation but over the first few hours of life developed a tachypnoea and lactic acidosis (9.4 mmol/l) requiring transfer to intensive care. On examination he was noted to be hypotonic and had hypospadias and an inguinal hernia. Echocardiograph revealed a mild hypertrophic cardiomyopathy. Clotting was deranged with a prolonged APTT of 120 s and Factor VIII activity was recorded at <1%, consistent with a diagnosis of Haemophilia A. Therapy with sodium bicarbonate and recombinant Factor VIII was commenced and he was discharged to outpatient follow-up. Neurodevelopmental progress was very slow and head growth remained poor. He developed a progressive spastic dystonic movement disorder with nystagmus. Weight gain was poor and he suffered a number of (aspiration) pneumonias. At age 4 years he was admitted to hospital in acute respiratory failure and during this admission a PEG (percutaneous endoscopic gastrostomy) was inserted. He died a few months later following another admission in acute respiratory failure complicated by bleeding.

#### Patient 9 (Family 6)

This boy was the first child of first cousin UK-Pakistani parents and was born at 36 weeks gestation, weighing 1780 g, following a labour complicated by foetal distress. He was dysmorphic with features of micrognathia, polysyndactyly and hypospadias. Renal impairment was noted on Day 2 and raised creatinine persisted throughout life. Ultrasound examination revealed small, hyperechoic kidneys with poor corticomedullary differentiation and a trabeculated bladder. Echocardiography showed a bicuspid aortic valve. Feeding was poor, necessitating supplemental feeding with a nasogastric tube. A persistent lactic acidosis of 4–5 mmol/l was noted and CSF lactate was 3.41 mmol/l. Muscle biopsy showed only mild fibre size variation, but with deficiency of Complex IV. He became increasingly unwell with repeated episodes of pneumonia and died at age 6 months.

#### Patient 10 (Family 7)

This girl is the second of two children of first cousin Iraqi parents and was born at term by emergency Caesarean section for foetal distress weighing 4150 g. No obvious facial dysmorphism was observed. At 6 months, developmental delay, muscular hypotonia, truncal ataxia and dysmetria were noticed. During viral infections the ataxia worsened and she lost motor skills. At best, the girl was able to walk with support. Serum lactate ranged between normal and 7.8 mmol/l and CSF lactate was 4.0 mmol/l. At age 23 months, following an acute upper respiratory tract infection, the girl developed constant esotropia of the right eye, significant motor regression, dysphagia, arterial hypertension and left ventricular hypertrophy. Dysphagia required PEG insertion. At age 24 months RSV bronchiolitis led to respiratory insufficiency and intermittent mechanical ventilation, followed by tracheostomy and long-term ventilation. At age 27 months, the girl is receiving palliative care at home.

### Neuroimaging results

**Figure 1:**
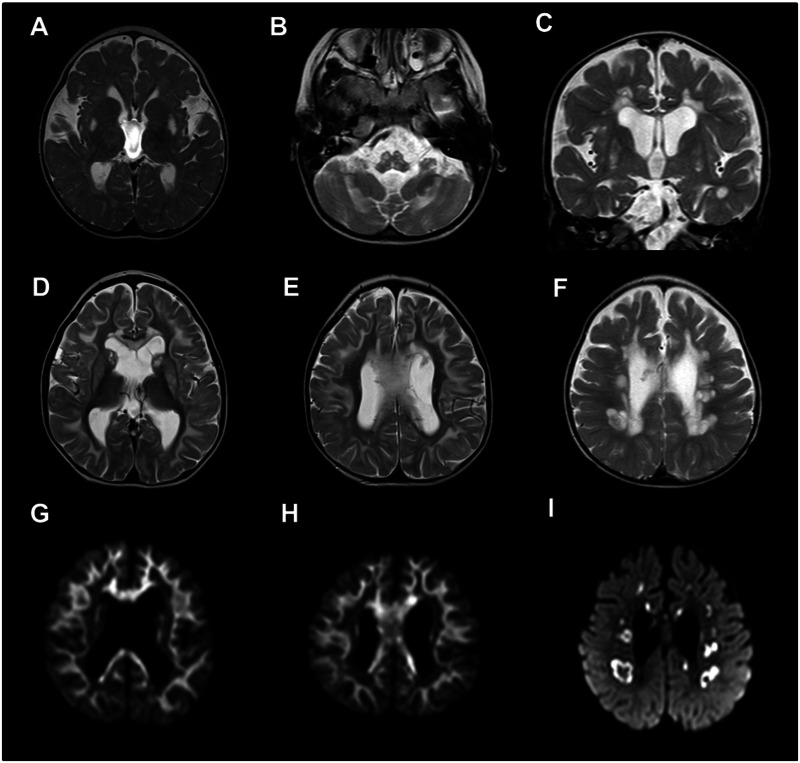
**Brain abnormalities in patients harbouring *LRPPRC* mutations identified by cranial magnetic resonance imaging**. (**A**) Axial T_2_ demonstrating symmetrical T_2_ hyperintensity in the putamina (Patient 8). (**B**) Axial T_2_ demonstrating symmetrical medullary and cerebellar hyperintensity (Patient 8). (**C**) Coronal T_2_ demonstrating symmetrical putaminal and periventricular T_2_ hyperintensity (Patient 10). Appearances in **A–C** are consistent with Leigh syndrome phenotype. (**D–F**) Axial T_2_ images of the brain with corresponding diffusion-weighted imaging sequences (**G–I**) demonstrating active demyelinating leucoencephalopathy involving the subcortical U-fibres, basal ganglia and corpus callosum in **D**, **E**, **G** and **H** (Patient 2) and cystic demyelination in the deep white matter in **F** and **I** (Patient 8).

Six of the 10 patients presented in this case series [Patients 1, 2, 4, 8 (on three occasions), 9 and 10] had cranial MRI scans, showing a range of abnormalities; four of six patients (Patients 1, 2, 4 and 9) demonstrated a spectrum of congenital abnormalities listed in detail in Table 1. There are known associations between mitochondrial disorders (e.g. pyruvate dehydrogenase deficiency) and dysgenesis of the corpus callosum, but this has not been previously described in association with *LRPPRC* mutation. Two of six patients (Patients 8 and 10) demonstrated symmetrical signal changes involving the brainstem on cranial MRI typical of the neuroradiological features of Leigh syndrome (Fig. 1A–C). A further two patients (Patients 1 and 2) showed symmetrical signal changes in the basal ganglia and cerebellum, but without evidence of brainstem involvement and their clinical course was not typical for Leigh syndrome. Three of the patients (Patients 2, 8 and 10) also demonstrated striking symmetrical leukoencephalopathy with avid restricted diffusion, a feature not previously documented in cases with *LRPPRC* mutation (Fig. 1D–F). In Patient 8, the rapidly progressive nature of this cystic leukoencephalopathy was further catalogued on imaging at 7, 8 and 11 months of age as shown in later scans in Fig. 1F and I. In addition, the single voxel proton magnetic resonance spectrum acquired on Patient 8 demonstrated elevated lactate (data not shown).

**Table 1:** Neuroimaging features identified in patients with *LRPPRC* mutations

Patient ID	Age at scan	Congenital abnormalities	Acquired abnormalities
Patient 1	1 year old	Partial agenesis of corpus callosum Hippocampal malformation	Abnormal signal observed in dentate nuclei and fastigial nuclei of the cerebellum and posterior putamina
Patient 2	4 years old	Mega cisterna magna Hypoplastic cerebellar vermis Hypoplastic cerebellar tonsils Unfolded hippocampi	Supratentorial central and cerebellar atrophySymmetrical abnormal T2 signal in corpus callosum, corpus striatum and supratentorial subcortical white matter (U fibres) – also restricts on DWISymmetrical abnormal signal of dentate nuclei but no DWI changesThalami spared
Patient 4	1 month old	Unfolded hippocampi	None reported
Patient 8	Scanned at 7, 8 and 11 months of age	None reported	Progressive central atrophy and ventricular dilatation Prominent cystic periventricular white matter changes including corpus callosum (sparing of U fibres) - avid restricted diffusion on DWI Symmetrical cerebellar white matter, putaminal and dentate nuclei and midbrain and medulla signal changes
Patient 9	3 months old	Simplified gyral pattern Under operculization of the sylvian fissures Bilateral frontal polymicrogyria Bilateral parieto-occipital pachygyria Low lying torcula	None reported
Patient 10	14 months old	None reported	Symmetrical signal changes involving the corpus callosum, spinocerebellar and inferior olivary tracts All associated with restricted diffusion

DWI = diffusion-weighted imaging.

### Skeletal muscle and fibroblast respiratory chain enzyme activities

Investigation of diagnostic muscle biopsies available from seven patients revealed severe and isolated histochemical COX defects (Patients 1, 2, 4, 5, 6, 8 and 9) and isolated Complex IV deficiency in muscle (Table 2), with normal activities of other respiratory chain complexes. Interestingly, we noted normal mitochondrial respiratory chain activities, including Complex IV, in fibroblasts from some patients (Patients 1 and 2) although a defect was observed in the cells from Patient 5, later confirmed to harbour the same genetic variant as Patients 1 and 2, and to a lesser extent in Patients 4 and 10 (Table 2).

**Table 2:** Clinical, molecular and biochemical characteristics of patients with *LRPPRC* mutations

Patient ID (Sex) Family	Family history	Country of origin	Age of onset/age at last follow-up	Clinical presentations								% Residual Complex IV Activity^c^	*LRPPRC* mutations
				Muscle	Heart	CNS	Cranial MRI	Developmental delay	Lactic acidosis	Dysphagia	Hypoglycaemia		
P1 (F) Family1	2nd cousins	UK-Pakistani	Birth/15 months^a^	+	+	+	+	+	+^b^	+	–	29(M); 70(F); uniform COX histochemical defect in muscle	Homozygous c.3900+1G>T: p.Arg1276_Lys1300del
P2 (M) Family 2	1st cousins	UK-Pakistani	8 months/8 years	+	–	+	+	+	+	+	–	3(M); 70(F); uniform COX histochemical defect in muscle	Homozygous c.3900+1G>T: p.Arg1276_Lys1300del
P3 (F) Family 2	1st cousins	UK-Pakistani	Birth/3 years	–	–	+	n.d.	+	+^b^	+	+	n.d.	Homozygous c.3900+1G>T: p.Arg1276_Lys1300del
P4 (M) Family 3	unrelated	UK-Caucasian	2 days/6 weeks^a^	+	–	+	+	–	+^b^	+	+	21(M); 30(F); uniform COX histochemical defect in muscle	c.[1582+7A>G];[3147dupA]: p.[=, Glu497*];[Gly1050Argfs*4]
P5 (F) Family 4	1st cousins	UK-Indian	1 day/3 years^a^	+	–	+	n.d.	+	+^b^	+	+	33(M); 14(F); uniform COX histochemical defect	Homozygous c.3900+1G>T: p.Arg1276_Lys1300del
P6 (F) Family 4	1st cousins	UK-Indian	Birth/9 days^a^	–	+	–	n.d.	–	+^b^	–	n.d.	41(M); uniform COX histochemical defect	Homozygous c.3900+1G>T: p.Arg1276_Lys1300del
P7 (F) Family 4	1st cousins	UK-Indian	Birth/9 weeks	–	–	–	n.d.	–	+^b^	+	+	n.d.	Homozygous c.3900+1G>T: p.Arg1276_Lys1300del
P8 (M) Family 5	1st cousins	Turkish	Birth/4 years^a^	+	+	+	+	+	+^b^	+	–	22(M); uniform COX histochemical defect	Homozygous c.2595_2597delGGT: p.(Val866del)
P9 (M) Family 6	1st cousins	UK-Pakistani	Birth/6 months^a^	–	+	+	+	–	+	+	–	33(M); uniform COX histochemical defect in muscle	Homozygous c.3900+1G>T: p.Arg1276_Lys1300del
P10 (F) Family 7	1st cousins	Iraqi	6 months/27 months	+	+	+	+	+	+	+	–	37(F)	Homozygous c.2726_2728delAGA: p.(Lys909del)

^a^ age at death; ^b^ lactic acidaemia at birth; ^c^ mitochondrial Complex IV activity is expressed as a percentage of control mean values for enzyme activity relative to citrate synthase activity as determined in skeletal muscle (M) and fibroblasts (F); n.d. = not determined.

### Whole exome sequencing identifies biallelic *LRPPRC* mutations

Mutations in the mitochondrial genome (mtDNA rearrangements and mtDNA point mutations) were excluded, as were mutations in a number of candidate nuclear genes involved in COX assembly including *SURF1*, *SCO1*, *SCO2*, *COX10* and *COX15* by Sanger sequencing.

**Figure 2:**
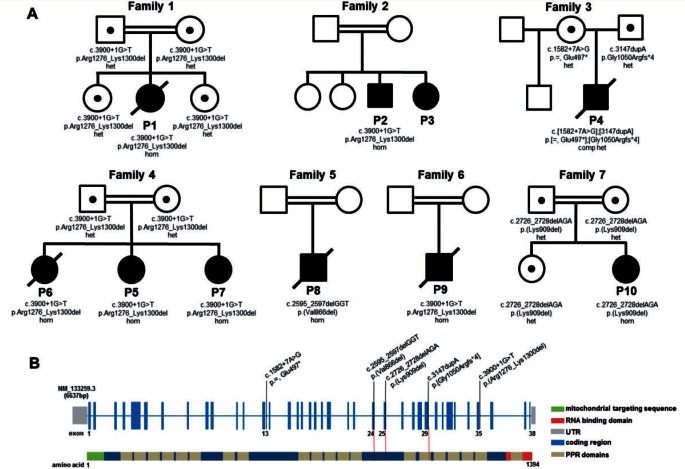
**Family pedigrees and *LRPPRC* mutations and gene structure.** (**A**) Pedigrees of the seven families with mutations in the *LRPPRC* gene. (**B**) A graphical representation of the *LRPPRC* gene structure illustrating mutated residues identified within the seven affected families. The coding regions are shown in blue and the predicted mitochondrial targeting sequence of the protein in green. The RNA binding domain is represented in red, while the 20 PPR domains are shown in beige.

DNA from six probands (Patients 1, 4, 5, 8, 9 and 10) was subjected to whole exome sequencing, filtering the raw data in a step-wise process to prioritize genes encoding proteins with a known or predicted mitochondrial localization harbouring rare, recessively-inherited (compound heterozygous or homozygous) variants (Mayr *et al.*, 2012; Bonnen *et al.*, 2013). This allowed us to identify causal mutations in the *LRPPRC* gene in all six patients, including the same homozygous c.3900+1G>T mutation in Patients 1, 5 and 9, which abolishes the consensus donor splice site of intron 35 leading to complete skipping of exon 35 as confirmed by cDNA studies (Supplementary Fig. 1A–C and Supplementary material); the homozygous c.3900+1G>T mutation was also identified in Patient 2 by candidate gene screening. Patient 4 harboured compound heterozygous mutations including a c.3147dupA frameshift mutation and a c.1582+7A>G mutation predicted, and subsequently confirmed, to lead to aberrant splicing (Supplementary Fig. 1D). Patient 8, the son of consanguineous first cousin Turkish parents harboured a novel c.2595_2597delGGT mutation, and Patient 10 harboured a novel c.2726_2728delAGA mutation. Where available, testing of parental samples confirmed carrier status and the analysis of DNA samples from affected siblings showed segregation of the mutant alleles with a clinical phenotype (Fig. 2).

### Loss of LRPPRC is associated with decreased respiration

**Figure 3:**
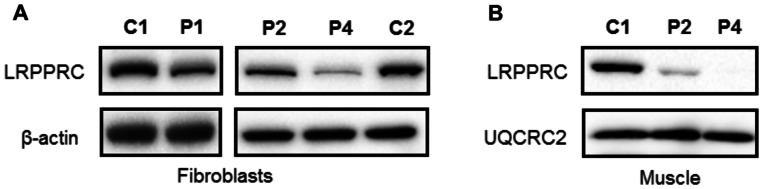
**Steady-state LRPPRC protein levels are reduced in LRPPRC patient tissues.** Western blot analysis of LRPPRC levels in protein extracts isolated from (**A**) fibroblasts and (**B**) skeletal muscle of control (C1, C2), homozygous *LRPPRC* (Patients 1 and 2; P1, P2) and compound heterozygous (Patient 4, P4) *LRPPRC* patients. In (**A**) β-actin and in (**B**) UQCRC2 antibodies were used as loading controls.

We investigated the biochemical phenotype of mutant LRPPRC derived from skin fibroblasts and muscle homogenates of two affected individuals with the homozygous c.3900+1G>T, p.(Gly1050Argfs*4) *LRPPRC* mutation (Patients 1 and 2) and a patient with c.[1582+7A>G];c.[3147dupA], p.[=,Glu497*];[Gly1050Argfs*4] compound heterozygous mutations (Patient 4). First, we determined the steady-state levels of LRPPRC in patients’ fibroblasts and muscle protein extracts by western blot analysis, confirming a marked decrease in the steady-state levels of LRPPRC protein in fibroblasts from all patients compared to age-matched controls (Fig. 3A). Mitochondrial protein extracts from muscle of Patients 2 and 4 also showed a marked decrease in the steady-state levels of the mutant LRPPRC protein (Fig. 3B).

**Figure 4:**
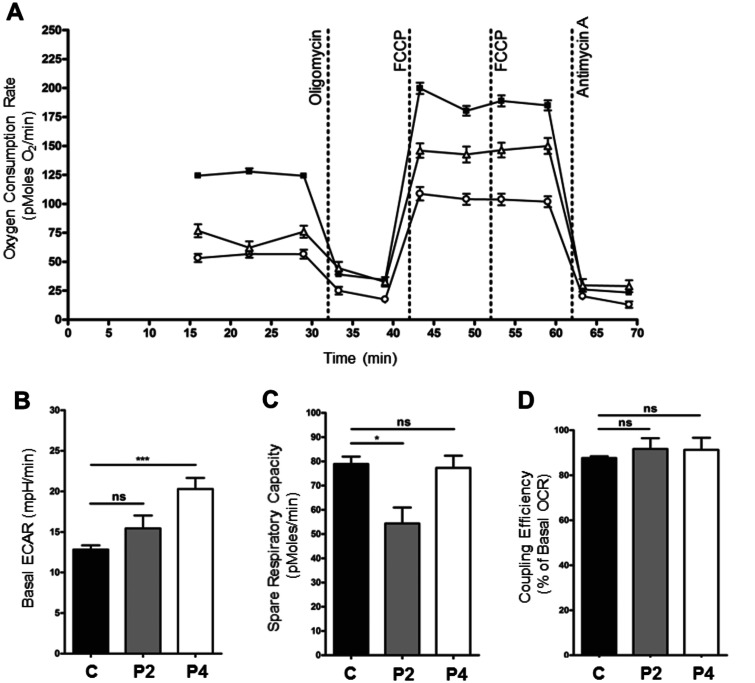
**Dysfunction of mitochondrial respiration in LRPPRC patient fibroblasts.** (**A**) Micro-scale oxygraphy analysis of live fibroblasts (XF24 Analyzer, Seahorse Biosciences) demonstrated a respiratory deficiency in cells from both the patient carrying the homozygous mutation (Patient 2, P2) (*n* = 17, open triangles) and the patient carrying the compound heterozygous mutation (Patient 4, P4) (*n* = 16, open circles) compared to control fibroblasts (*n* = 160, filled squares). Both basal (prior to oligomycin injection) and maximal (post-FCCP injection) OCR were significantly reduced (*P* < 0.05). (**B**) Basal ECAR (extra-cellular acidification rate), a measure of glycolysis in fibroblasts from Patient 4 was significantly increased in comparison to controls. The basal ECAR in fibroblast derived from Patient 2 was raised but not significantly different to controls. (**C**) The spare respiratory capacity (maximal OCR minus basal OCR) of Patient 2 fibroblasts was significantly decreased in comparison to controls; however, the spare respiratory capacity in Patient 4 fibroblasts was unchanged. (**D**) The coupling efficiency of respiration (oligomycin-sensitive OCR as per cent of basal OCR) was not changed in Patient 2 and Patient 4 fibroblasts compared to controls.

To further characterize the biochemical phenotype in LRPPRC patients, OCR were measured in patients’ fibroblasts. A significant decrease in basal OCR was observed in Patients 2 and 4 fibroblasts when compared to the control cell line (Fig. 4A). In addition, basal ECAR (extra-cellular acidification rate), a measure of glycolysis, was significantly increased in fibroblasts from Patient 4 in comparison with the controls, suggesting that patient fibroblasts are compensating for reduced ATP synthesis resulting from decreased mitochondrial respiration through increased glycolysis (Fig. 4B). Interestingly, however, basal ECAR in fibroblasts from Patient 2 was raised but not significantly different to controls (Fig. 4B). The spare respiratory capacity of Patient 2 fibroblasts was significantly reduced in comparison to control, suggesting that the mitochondria are working at near-maximal capacity, with little capability to respond to increased energy demand. However, Patient 4 fibroblasts showed an unchanged spare respiratory capacity (Fig. 4C). The efficiency of the coupling of respiration and ATP synthesis (oligomycin-sensitive OCR as a per cent of basal OCR) was unchanged in patient fibroblasts compared to controls, indicating normal levels of proton leak across the membrane (Fig. 4D). Importantly, patient cells containing reduced levels of LRPPRC protein display a biochemical phenotype, the nature of which was further investigated.

### Steady-state levels of respiratory chain components and complexes

**Figure 5:**
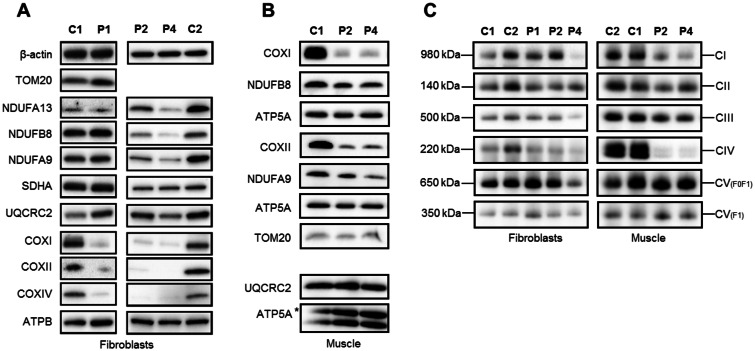
**Steady-state levels of OXPHOS components and complexes.** Western blot analysis of subunits of the respiratory chain complexes in (**A**) cell lysates of fibroblasts and (**B**) mitochondrial extracts of muscle isolated from control (C1, C2), homozygous *LRPPRC* (Patient 1, P1; Patient 2, P2) and compound heterozygous *LRPPRC* (Patient 4, P4) patient samples. Subunit-specific antibodies were used against CI (NDUFA13, NDUFA9, NDUFB8), CII (SDHA), CIII (UQCRC2), CIV (COXI, COXII, COXIV) and CV (ATP5A, ATPB). (**A** and **B**) LRPPRC patient fibroblasts and muscle show an almost complete loss of respiratory chain subunits from Complex IV, as well as significant loss of some subunits from Complex I. Complex III showed a mild loss of subunit UQCRC2 in Patient 4 fibroblasts, while Complex V showed no loss of protein in either patient fibroblasts or muscle samples. The nucleus-encoded Complex II (SDHA), cytosolic β-actin and the outer mitochondrial membrane marker TOM20 were used as loading controls. (**C**) Mitochondrial proteins from fibroblasts and skeletal muscle samples of control (C1, C2) and homozygous *LRPPRC* (Patients 1 and 2) and compound heterozygous *LRPPRC* (Patient 4) patients were extracted in DDM and analysed by one-dimensional Blue Native PAGE. Subunit-specific antibodies [CI (NDUFA9), CII (SDHA), CIII (UQCRC2), CIV (COX1) and CV (ATP5A)] were used to assess the assembly of individual OXPHOS complexes. Immunoblot analysis revealed decreased amounts of fully assembled Complex IV in patient fibroblasts and muscle compared to age-matched controls. A decrease in Complex I assembly was observed in LRPPRC patients muscle (Patients 2 and 4) and compound heterozygous fibroblasts (Patient 4). Complex II (SDHA) was used as a loading control.

The steady-state protein levels of subunits of mitochondrial respiratory chain complexes in LRPPRC patient samples were analysed by SDS-PAGE and immunoblotting. Homozygous mutant *LRPPRC* (Patients 1 and 2) and compound heterozygous mutant *LRPPRC* (Patient 4) fibroblasts showed an almost complete loss of mitochondrial (COXI and COXII) and nuclear-encoded (COXIV) subunits of Complex IV (Fig. 5A). The steady-state levels of Complex I subunit proteins NDUFB8, NDUFA9 and NDUFA13 were also decreased in Patients 2 and 4 fibroblasts. The levels of mitochondrial encoded Complex III subunit UQCRC2 were markedly decreased in Patient 4 fibroblasts compared to control cell lines, but normal or increased in the homozygous LRPPRC patient fibroblasts (Patients 1 and 2) (Fig. 5A). Interestingly, protein loss in each case was more severe in Patient 4, perhaps reflecting the more severe compound heterozygous frameshift and splice-site mutations. The nuclear-encoded subunits of Complex II and Complex V did not show any loss of protein (Fig. 5A).

Consistent with the reduced steady-state levels of Complex IV subunits in LRPPRC patient fibroblasts, mitochondrial protein extracts from muscle homogenates also revealed a significant decrease in the steady-state levels of COXI and COXII (Fig. 5B). In addition, slightly decreased levels of Complex I subunit proteins NDUFB8 and NDUFA9 were found in LRPPRC patients’ muscle homogenates, whilst the levels of Complex V and Complex III subunits remained unchanged (Fig. 5B).

The assembly of OXPHOS complex subunits into mitochondrial respiratory chain complexes was subsequently analysed by blue native PAGE in LRPPRC patient fibroblasts and skeletal muscle. Blue native PAGE analysis showed a slight decrease of fully assembled Complex IV in Patient 1 and 2 fibroblasts carrying the homozygous c.3900+1C>T mutation and an almost complete lack of Complex IV in the muscle of Patient 2 (Fig. 5C). Additionally, LRPPRC patient muscle homogenates displayed decreased levels of Complex I, similar to previously published studies (Fig. 5C) (Sasarman *et al.*, 2015). The amounts of fully assembled Complexes I, III, IV and V were markedly decreased in Patient 4 fibroblasts, likely due to the more severe nature of the compound heterozygous *LRPPRC* gene mutations. Interestingly however, this was not the case in Patient 4’s muscle homogenates, where the assembly profile of Complex III and Complex V was normal.

### The absence of LRPPRC affects the stability of mitochondrial transcripts

**Figure 6:**
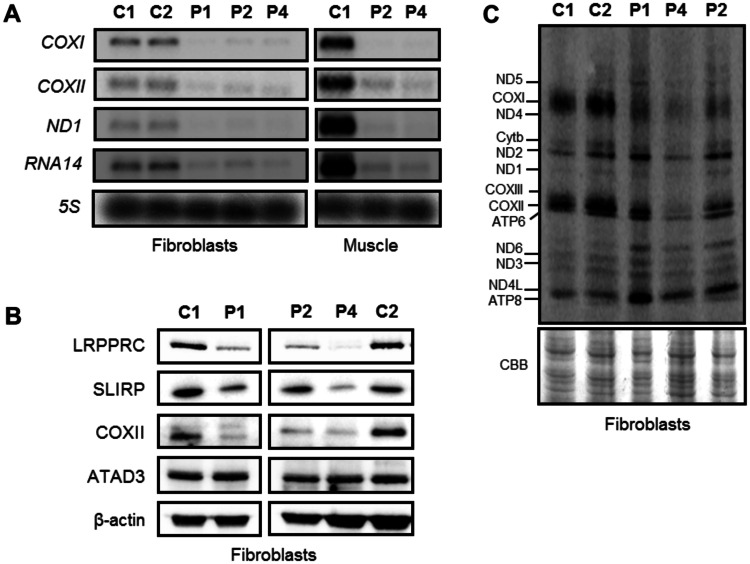
**The effect of *LRPPRC* mutations on the steady-state levels of mitochondrial mRNAs and mitochondrial translation products.** (**A**) Mitochondrial mRNAs in fibroblasts and skeletal muscle from LRPPRC patients were significantly reduced. Total RNA was isolated from control and patient fibroblasts and muscle, and the levels of mitochondrial mRNAs were analysed by northern blotting. Probes against specific mitochondrial open reading frames, *COXI*, *COXII*, *ND1* and *RNA14* were used and a probe for the *5S* cytosolic rRNA was used as loading control. (**B**) Reduction in SLIRP steady-state protein levels in the LRPPRC patient fibroblasts were confirmed by western blot analysis. β-Actin was used as loading control. (**C**) Analysis of mitochondrial-encoded polypeptides by ^35^S methionine/cysteine pulse labelling revealed a marked decrease in mitochondrial translation in the compound heterozygous patient cells (Patient 4) compared to age-matched controls (C1 and C2). Coomassie Brilliant Blue (CBB) staining was used to check for equal loading of the samples.

Loss of LRPPRC has been previously associated with a decrease in the steady-state levels of mitochondrial mRNA transcripts (Sasarman *et al.*, 2010, 2015; Bratic *et al.*, 2011; Ruzzenente *et al.*, 2012). We investigated the effect of *LRPPRC* mutations on the stability of mitochondrial mRNAs in fibroblasts and skeletal muscle samples from control and LRPPRC patients. In both tissues we detected decreased steady-state levels of *MTCO1*, *MTCO2*, *MTND1* and *RNA14* transcripts (Fig. 6A). These data are in agreement with previously published work showing decreased mitochondrial transcript levels in tissues derived from LSFC patients (Sasarman *et al.*, 2010, 2015) and *LRPPRC* knockdown cell lines (Cooper *et al.*, 2008). Studies in humans and mice have shown that LRPPRC forms a ribonucleoprotein complex with SLIRP and this complex protects the non-translated mitochondrial mRNA transcripts (Sasarman *et al.*, 2010; Chujo *et al.*, 2012; Ruzzenente *et al.*, 2012). Loss of SLIRP mimics the post-transcriptional defect observed in LSFC patient fibroblasts, thus highlighting their functional dependence. As expected the steady-state protein levels of SLIRP decreased and reflected the levels of LRPPRC in patient fibroblasts (Fig. 6B).

In addition to its role in the regulation of mRNA stability, LRPPRC has been also implicated in the regulation of mitochondrial mRNA polyadenylation (Bratic *et al.*, 2011; Chujo *et al.*, 2012; Ruzzenente *et al.*, 2012; Wilson *et al.*, 2014). Therefore, we assessed any changes in the length of the poly(A) tail in the mitochondrial *RNA14* transcript in the homozygous (Patient 2) and compound heterozygous (Patient 4) LRPPRC fibroblasts using a poly(A) tail length assay (Temperley *et al.*, 2010). Analysis of the mitochondrial *RNA14* transcript showed that polyadenylation in both Patients 2 and 4 fibroblasts was similar to the control cell line (Supplementary Fig. 2). In addition, we found that the steady-state levels of the poly(A) polymerase interacting protein ATAD3 (encoded by *ATAD3A*) were also normal in LRPPRC patient skin cell lines (Fig. 6B) (He *et al.*, 2012).

To determine whether the observed reduction in steady-state levels of mitochondrial mRNAs caused by mutated LRPPRC, affected the translation of mitochondrially-encoded proteins, ^35^S-methionine/cysteine pulse labelling was performed. Patients 1 and 2, harbouring the homozygous c.3900+1G>T mutation, showed a mild decrease in the synthesis of COX1, whereas the translation of other mtDNA-encoded polypeptides was the same or slightly increased compared to control fibroblasts (Fig. 6C). A severe decrease in the *de novo* protein synthesis of almost all mitochondrial-encoded polypeptides was observed in Patient 4 (Fig. 6C).

## Discussion

The occurrence of LSFC is common to the Saguenay-Lac-Saint-Jean region of Québec, with a carrier frequency estimated at 1/23 inhabitants in Saguenay-Lac-Saint-Jean, with 1 in 2000 individuals being affected (Morin *et al.*, 1993). LSFC is caused by a founder mutation in the *LRPPRC* gene: in a study of 56 patients, 55 were homozygous for the c.1061C>T, p.(Ala354Val) mutation, while one individual was compound heterozygous for this mutation in *trans* with a c.3830_3837del, p.(Cys1277*) mutation (Debray *et al.*, 2011). Using a whole-exome sequencing approach, we have identified novel pathogenic *LRPPRC* mutations in 10 patients from outside the French-Canadian population. None of these carried the p.(Ala354Val) founder mutation. Instead, we found three novel homozygous mutations in *LRPPRC*: c.3900+1G>T, p.(Arg1276_Lys1300del); c.2595_2597delGGT, p.(Val866del) and c.2726_2728delAGA, p.(Lys909del); and a genetic compound c.[1582+7A>G];[3147dupA], p.[=,Glu497*];[Gly1050Argfs*4]. Of these, p.(Arg1276_Lys1300del) may be relatively common in the Indian subcontinent: it was found in all four families who originated from there, although they were unrelated and came from different regions.

Our patients’ clinical features resembled LSFC in many respects. As a group, however, our patients were more severely affected; presumably this reflects the severity of their mutations compared with the founder mutation in LSFC. Thus, all our patients had severe neurodevelopmental impairment; the least affected (Patient 5) having no language acquisitions. Most of our patients have had episodes of neurological deterioration, as described in LSFC; Patients 2 and 3 have been stable for a number of years, resembling older LSFC patients. Neurological problems in our patients have included motor difficulties (hypotonia, dystonia or ataxia), dysphagia, strabismus and seizures — as in LSFC. Neuroimaging in two of our patients showed the appearances of Leigh syndrome, as in LSFC, but the characteristic brainstem lesions were absent in other patients. Moreover, three of our patients had a striking leukoencephalopathy and four patients showed cerebral malformations, which have not been reported in LSFC (Table 1).

Episodes of severe, sometimes fatal, lactic acidosis are a prominent feature of LSFC, seldom seen in patients with other forms of Leigh syndrome (Debray *et al.*, 2011; Wedatilake *et al.*, 2013). Similar episodes of lactic acidosis occurred in many of our patients. The acidosis was often accompanied by ketosis but there was no hyperglycaemia, unlike the LSFC patients. Seven of our 10 patients had episodes of severe lactic acidosis within a few days of birth, often within the first few hours. In contrast, only 6 of 36 patients with LSFC had neonatal episodes of severe acidosis and the median age of presentation was 5 months (Debray *et al.*, 2011).

In LSFC, symptoms are limited to the brain and the episodes of acidosis, but a number of our patients had additional problems. For example, two of our patients had mild hypertrophic cardiomyopathy, a feature not previously observed in LSFC. More significantly, four of our patients had other congenital malformations, in addition to the cerebral malformations mentioned previously. One patient had complex congenital heart disease, another had bilateral superior vena cava and a third had a bicuspid aortic valve. Other malformations included hypospadias (in two of the four reported males), an anteriorly placed anus and polysyndactyly. Malformations have not been reported in LSFC patients, though they often have distinctive facial features, with a prominent forehead, broad nose and mild hirsutism. Similar facial features were noted in a few of our patients.

An intriguing aspect of our findings relates to the mitochondrial respiratory chain defects reported in patients with *LRPPRC* mutation. Previously, biochemical investigation of COX enzyme activity in patients with LSFC and a founder *LRPPRC* mutation revealed normal Complex IV activity in kidney and heart, 50% of control activities in fibroblasts and skeletal muscle and a severe COX enzyme defect in liver and brain (Merante *et al.*, 1993; Sasarman *et al.*, 2010, 2015). Surprisingly, the residual Complex IV activity in fibroblasts was only decreased in three of five patients we were able to test (Table 2) and did not appear to correlate with the molecular abnormality. The OXPHOS defect in *LRPPRC* patients is unlikely therefore to be simply related to Complex IV deficiency, and muscle samples from the homozygous p.(Arg1276_Lys1300del) and the genetic compound p.[=,Glu497*];[Gly1050Argfs*4] showing a combined Complex I and Complex IV respiratory chain assembly defect (Fig. 5) further support these data. In addition, our observation is in agreement with previous studies by Sasarman *et al.* (2015) that demonstrated differences in OXPHOS activity and assembly between distinct tissues in LSFC patients. Interestingly, *LRPPRC* patient fibroblasts harbouring the compound heterozygous p.[=,Glu497*];[Gly1050Argfs*4] mutation showed a more striking defect in the assembly of all OXPHOS complexes with the exception of Complex II. Further differences were observed in the steady-state levels of LRPPRC and OXPHOS subunits, which were markedly decreased, compared to other patients. This may reflect the more severe compound heterozygous frameshift and splice-defect nature of the mutations that make the product vulnerable to nonsense-mediated mRNA decay and subsequently affect the steady-state levels of LRPPRC protein. Indeed, Sasarman *et al.* (2010) showed that the steady-state levels of LRPPRC determine the extent of the OXPHOS deficiency. The decrease in the steady-state LRPPRC protein levels in the homozygous p.(Arg1276_Lys1300del) patients is to a lesser extent compared to the compound heterozygous LRPPRC patient. It is likely that the predicted effect of the homozygous p.(Arg1276_Lys1300del) in-frame deletion mutation may affect the overall stability of the LRPPRC protein, which may explain why the reduction in LRPPRC levels is not as severe for Patients 1 and 2 compared to Patient 4.

Unexpectedly, biochemical studies suggest that homozygous p.(Arg1276_Lys1300del) *LRPPRC* patient fibroblasts only show an isolated Complex IV deficiency, despite the reduction in the steady-state levels of both Complex I and Complex IV polypeptides. It has previously been suggested that the instability of Complex I subunits may activate a compensatory mechanism in the form of supercomplex formation, thus allowing the formation of an active Complex I (Calvaruso *et al.*, 2012); it remains to be determined whether supercomplex formation in the LRPPRC mutant cells triggers such an adaptive response mechanism but is an avenue for further research. Previous studies have also shown that in Complex I-deficient *Ndufs4* knockout mice, Complex III plays a major role in the stabilization of Complex I (Calvaruso *et al.*, 2012). Moreover, loss of the *Caenorhabditis elegans* homologue of the mammalian *LRPPRC* gene, *mma-1*, also showed a 50% reduction in the levels of Complex I subunits while Complex I activity remained unaffected (Rolland *et al.*, 2013). Mitochondrial hyperfusion appears to be responsible for the maintenance of mitochondrial function in these animals. Interestingly, the mitochondrial network in SH-SY5Y cells transfected for 3 days with *LRPPRC* siRNA remains stable and hyperfused. However, after 5 days, mitochondrial fragmentation occurs leading to a marked decrease in cellular ATP production (Rolland *et al.*, 2013). Fibroblasts from patients with LSFC have also been shown to manifest fragmented mitochondrial network (Sasarman *et al.*, 2010; Burelle *et al.*, 2015). It is, therefore, not unreasonable to hypothesize that Complex IV deficiency triggers an initial compensatory hyperfusion mechanism in LSFC patients’ fibroblasts, which subsequently fails, leading to fragmented mitochondrial network (Sasarman *et al.*, 2010; Rolland *et al.*, 2013).

The precise molecular mechanism through which LRPPRC stabilizes mitochondrial transcripts remains unclear. However, studies have shown that in order to stabilize the majority of polyadenylated mitochondrial mRNAs, LRPPRC forms a RNA-dependent protein complex with SLIRP that protects the mRNAs from 3’ exoribonuclease digestion (Sasarman *et al.*, 2010, 2015; Chujo *et al.*, 2012; Ruzzenente *et al.*, 2012). The reduction in LRPPRC steady-state levels found in affected patients’ fibroblasts and muscle correlates with a decrease in SLIRP protein levels (Fig. 6B) and these findings support the LRPPRC and SLIRP interdependence in the regulation of mitochondrial post-transcriptional mechanisms. Paradoxically, our data suggest that despite the reduced stability of mitochondrial *MTND1* and *RNA14* transcripts in homozygous p.(Arg1276_Lys1300del) *LRPPRC* fibroblasts, no obvious changes in the translation of the encoded mitochondrial polypeptides were found and the assembly of Complex I and Complex V was normal. A global reduction in the steady-state levels of mitochondrial mRNAs was also found in LSFC fibroblasts, yet the synthesis of some mitochondrial polypeptides was disproportionate, with the exception of COX subunits (Sasarman *et al.*, 2010). It remains to be determined why the synthesis of some mitochondrial proteins is unaffected despite the global decrease in steady-state levels of mitochondrial mRNAs in *LRPPRC* patient cells. LRPPRC appears to be necessary for polyadenylation of mitochondrial mRNAs (Chujo *et al.*, 2012; Ruzzenente *et al.*, 2012; Wilson *et al.*, 2014), which can modulate transcript stability (Wydro *et al.*, 2010). To date there are no studies describing the length of the poly(A) tail of mitochondrial transcripts in *LRPPRC* patients. We show that the poly(A) tail of the mitochondrial *RNA14* mRNA was unaffected in both homozygous and compound heterozygous *LRPPRC* mutant individuals. Moreover, we did not observe any significant changes in the length of the poly(A) tail of mitochondrial transcripts *MTCO1, MTND3* or *RNA14* in cells depleted of LRPPRC or SLIRP (Chrzanowska-Lightowlers, unpublished data). Further studies will be required to determine whether the polyadenylation status of different mitochondrial mRNAs in patients carrying *LRPPRC* mutations is affected.

In conclusion, we have characterized the clinical and molecular nature of novel *LRPPRC* variants identified in 10 patients with early-onset COX deficiency associated with multi-organ involvement. Molecular diagnosis of mitochondrial disorders using next generation sequencing has allowed us to identify new mutations within *LRPPRC* gene outside of the French-Canadian population, affirming *LRPPRC* as a candidate gene in patients with early-onset COX deficiency and neurological deficits from different ethnic backgrounds.

## Funding

This work was supported by a Wellcome Trust Strategic Award (096919/Z/11/Z), the MRC Centre for Neuromuscular Diseases (G0601943), the Lily Foundation, the UK NHS Highly Specialised “Rare Mitochondrial Disorders of Adults and Children” Service in Newcastle upon Tyne and Oxford, the BMBF funded German Network for Mitochondrial Disorders (mitoNET #01GM1113C/D), E-Rare project GENOMIT (01GM1207), Juniorverbund in der Systemmedizin “mitOmics” (FKZ 01ZX1405C), an NIHR/CSO Healthcare Science Research Fellowship from the National Institute for Health Research (NIHR-HCS-D12-03-04) (to CLA) and by the Department of Health via the NIHR comprehensive Biomedical Research Centre award to Guy’s and St. Thomas’ NHS Foundation Trust in partnership with the King’s College London.

## Supplementary material

Supplementary material is available at *Brain* online.

Supplementary material

## Abbreviations

COX: cytochrome *c* oxidase
LSFC: French-Canadian variant of Leigh syndrome
OCR: oxygen consumption rate
OXPHOS: oxidative phosphorylation
PAGE: polyacrylamide gel electrophoresis
