# Supplementary material for: LRPPRC mutations cause early-onset multisystem mitochondrial disease outside of the French-Canadian population
Source: Brain. 2015 Oct 28;138(12):3503–19. doi: 10.1093/brain/awv291 (PMC4655343; doi:10.1093/brain/awv291)
Supplement: Supplementary material [file 91c117062ddced6166438033bf14809c_brain-2015-00976-File010.pdf]

SUPPLEMENTARY MATERIALS and METHODS:

Haplotype analysis

To determine whether the c.3900+1G>T mutation could be attributed to a common founder event, Patients 1, 2, 5 and 9 were genotyped for intragenic microsatellite markers (D2S4009 and D2S2298) and flanking microsatellite markers located upstream (D2S4023, D2S2182, D2S1247, D2S123 and D2S2251) and downstream (D2S119, D2S2259, D2S2272, D2S2220 and D2S2186) of the *LRPPRC* gene. Five of the microsatellite markers (D2S4023, D2S4009, D2S2298, D2S119 and D2S2259) revealed a common haplotype of at least 1.2Mb between all 4 patients, strongly suggesting that the c.3900+1G>T mutation could represent a common founder mutation within the UK-Asian population.

Molecular analysis of the splicing mutations

Total RNA was extracted from cultured fibroblasts from P1 using the ReliaPrep RNA Cell Miniprep System (Promega) and reverse-transcribed using the GoScript Reverse Transcription System (Promega), according to the manufacturer's guidelines. The resulting cDNA was PCR-amplified across the relevant regions using exonic primers and separated by agarose gel electrophoresis using standard conditions. PCR products were Sanger sequenced and data was analysed as described above.

For the transcript analysis of P4, RNA was prepared from cultured fibroblasts using the HP RNA isolation kit (Roche) and converted to cDNA with the AffinityScript Multiple Temperature cDNA synthesis kit (Agilent). The *LRPPRC* coding region was amplified in three overlapping segments and these were sequenced in both directions (primer sequences provided on request). Mutations were confirmed by sequencing the relevant section(s) of the gene in genomic DNA prepared from the cells with a Nucleon BACC2 kit GE Healthcare Life Sciences). To suppress nonsense mediated decay, near-confluent fibroblast cultures were

incubated in emetine (Sigma) at a final concentration of 100 μg/ml in DMEM/F12 medium with 10 % fetal calf serum, 100 U/ml penicillin and 100 μg/ml streptomycin for 7 hours before harvesting for RNA preparation.

Immunoblotting

The individual OXPHOS subunits and native complexes were visualised with the following primary antibodies: NDUFA9 (Molecular Probes, A21344), NDUFB8 (Abcam, ab110242), NDUFA13 (Abcam, ab110240), SDHA (MitoSciences, MS204), UQCRC2 (Abcam, ab14745), COX1 (Abcam, ab14705), COX2 (Molecular probes, A6404), COX4 (Abcam, ab14744), ATP5A (Abcam, ab14748), ATPB (Abcam, ab14730) and TOM20 (Santa Cruz, sc11415). Human LRPPRC, SLIRP, ATAD3 and β-actin proteins were detected with LRPPRC (Santa Cruz, sc66844), SLIRP (Abcam, ab51523), ATAD3 (kind gift from Dr. Ian Holt) and β-actin (Sigma, A1978) antibodies respectively. Species appropriate secondary horseradish peroxidise-conjugated antibodies were used (Dako, P0260 and P0399 respectively). The chemiluminescence signal was detected using ECL Prime Kit (GE Healthcare) and ChemiDocMP Imaging System (Bio-Rad).

Poly(A) tail assay

Total RNA was extracted using TRIZOL reagent (Invitrogen) following manufacturer's instructions. The mitochondrial poly(A) tail assay was performed essentially as described by Wilson and colleagues (Wilson *et al.*, 2014).

SUPPLEMENTARY FIGURES:

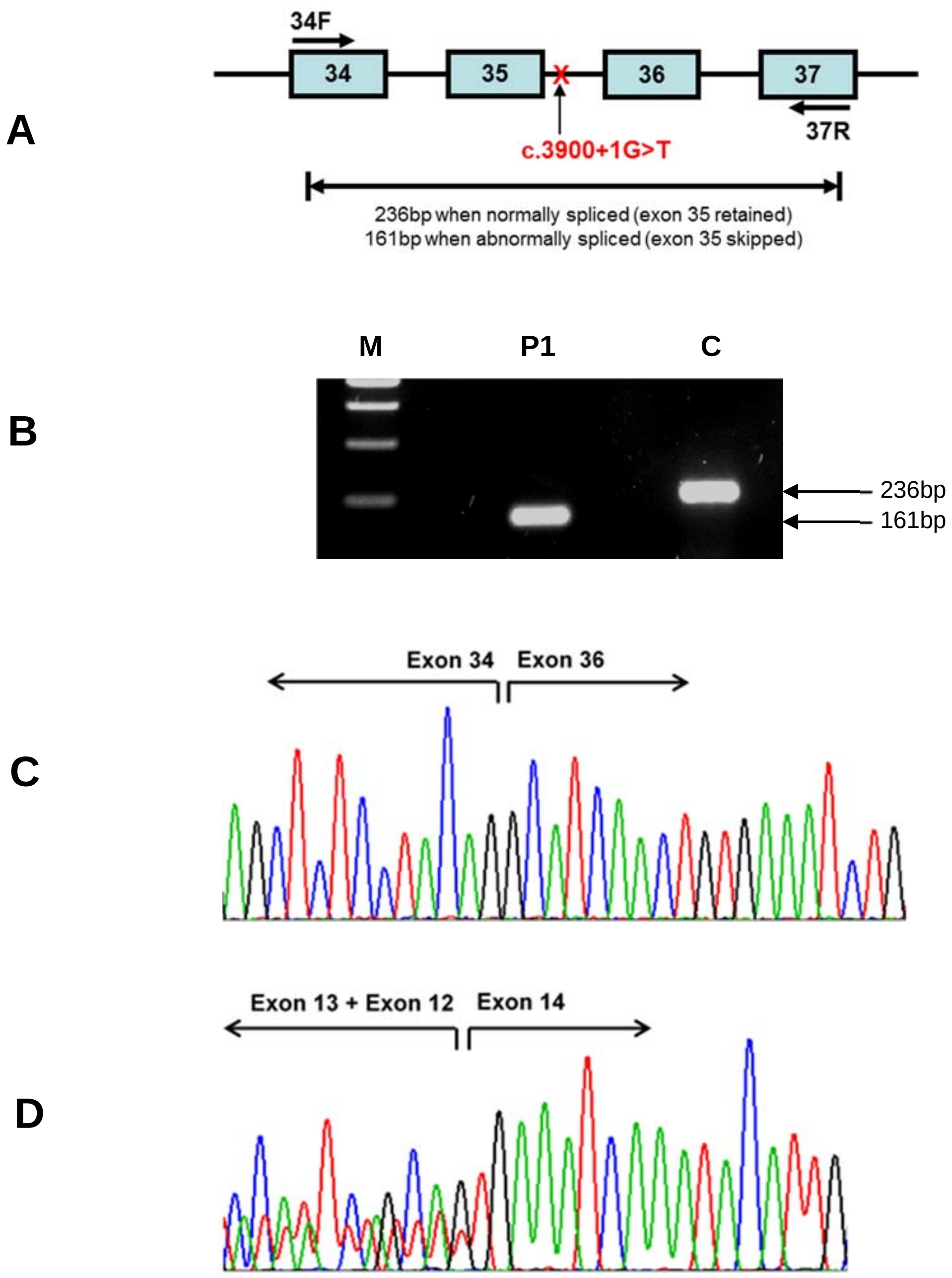

Supplementary Figure 1. Molecular analysis of the novel c.3900+1G>T and c.1582+7A>G *LRPPRC* splicing mutations.

[A] Schematic diagram showing the location of primers and the novel c.3900+1G>T splicing mutation within the *LRPPRC* gene. **[B]** Agarose gel electrophoresis of amplified cDNA from P1 and a tissue-matched normal control (C) alongside a molecular weight marker (M), with

the position of the normally spliced (236bp) and aberrantly-spliced (161bp) products indicated. **[C]** Sequencing of the abnormally-spliced product from P1 reveals that homozygosity for the novel c.3900+1G>T splicing mutation results in complete skipping of exon 35 from *LRPPRC* transcripts. The c.3900+1G>T splicing mutation was also confirmed in Patients 3, 5, 6 and 7 and 9. **[D]** cDNA was prepared from emetine-treated fibroblasts from P4. The sequence in the region of the splicing mutations in the patient is shown. P4 is heterozygous for a splicing mutation, c.1582+7A>G, which results in skipping of exon 13, however this is not seen unless the cells are treated with emetine to block nonsense-mediated decay. In this patient, a second mutation, an insertion of an A after position c.3147, also leads to nonsense-mediated decay of the derived transcript.

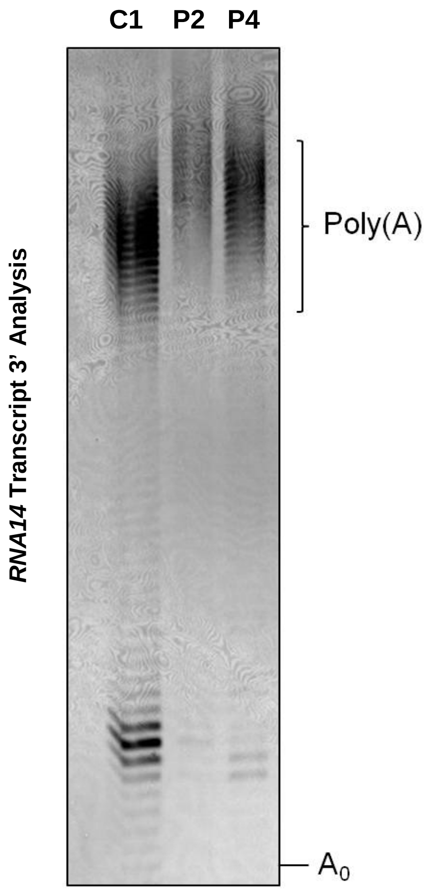

Supplementary Figure 2. Mitochondrial polyadenylation profile of *RNA14* transcript.

RNA isolated from control (C1), homozygous (P2) and compound heterozygous (P4) *LRPPRC* patients skin fibroblasts showed no changes in the length of the *RNA14* poly(A) tail. The position of the poly(A) tail is indicated in the upper panel and A_0 shows the start position of the post-3' processing of the mRNA.
