# Supplementary figures and images for: LRPPRC mutations cause early-onset multisystem mitochondrial disease outside of the French-Canadian population
Source: Brain. 2015 Oct 28;138(12):3503–19. doi: 10.1093/brain/awv291 (PMC4655343; doi:10.1093/brain/awv291)

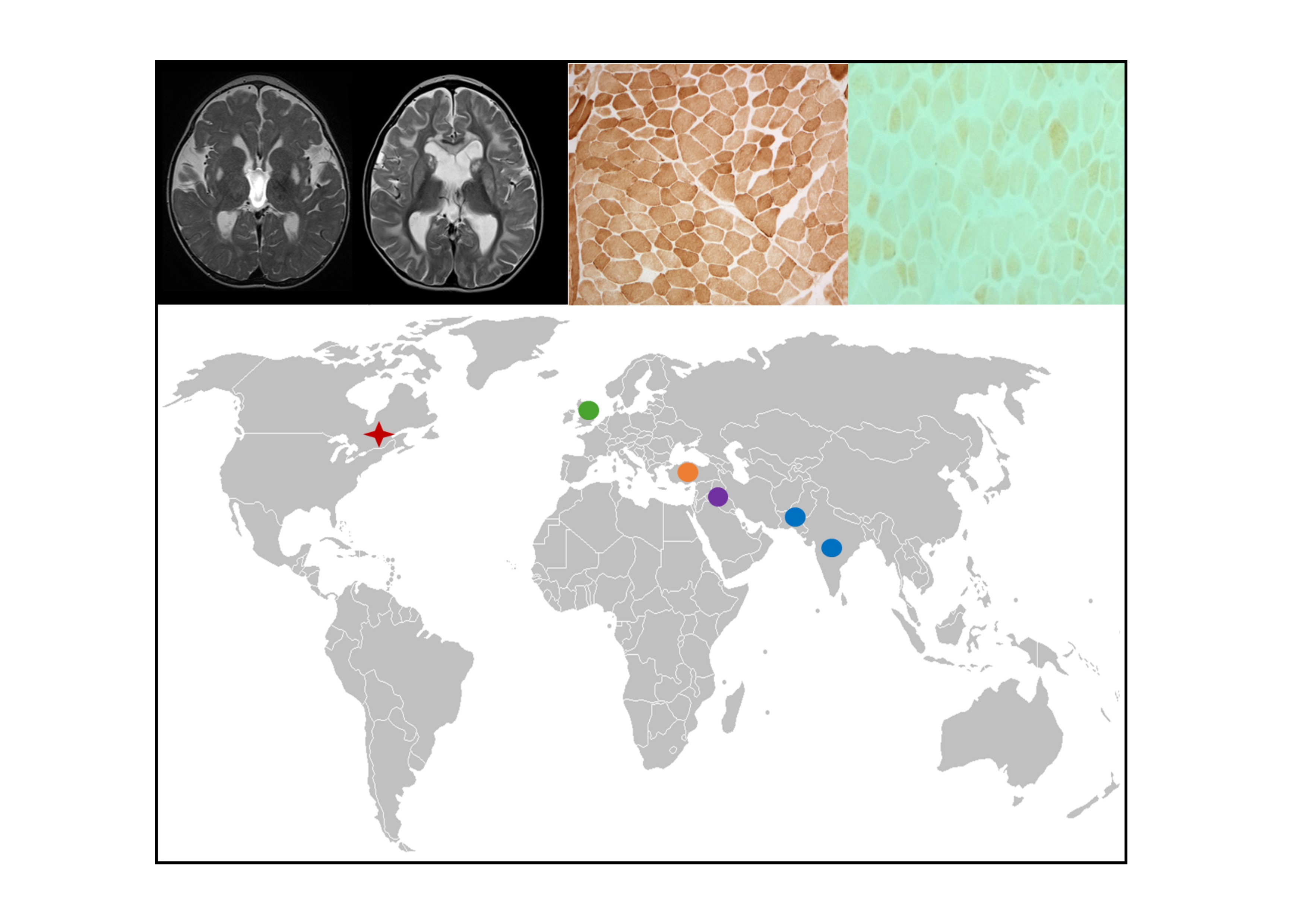

Supplement: Supplementary material [file c076cb00855e24ba0fb052078aefc1b6_brain-2015-00976-File011.jpg]
